# Strategies for assembling columns and layers in the *Drosophila* visual system

**DOI:** 10.1186/s13064-018-0106-9

**Published:** 2018-06-07

**Authors:** S. Sean Millard, Matthew Y. Pecot

**Affiliations:** 1 School of Biomedical Sciences, The University of Queensland, Brisbane, QLD 4072 Australia; 2 Department of Neurobiology, Harvard Medical School, Boston, MA 02115 USA

**Keywords:** *Drosophila* visual system, Layers, Columns, Columnar restriction, Layer specificity, Synaptic specificity

## Abstract

A striking feature of neural circuit structure is the arrangement of neurons into regularly spaced ensembles (i.e. columns) and neural connections into parallel layers. These patterns of organization are thought to underlie precise synaptic connectivity and provide a basis for the parallel processing of information. In this article we discuss in detail specific findings that contribute to a framework for understanding how columns and layers are assembled in the *Drosophila* visual system, and discuss their broader implications.

## Background

The patterning of neural connections into columns and layers is a hallmark of neural connectivity in complex nervous systems. These structural motifs are prevalent within vertebrates and invertebrates and underlie neural circuit organization in diverse regions including the insect optic lobe, and the cerebral cortex in mammals. The widespread use of these arrangements, and the characteristic columnar and layer patterns exhibited by specific neuron types suggests that this organization is of fundamental importance to nervous system function. Thus, elucidating general molecular and cellular principles underlying how neurons organize into columnar and layered networks is central to understanding how nervous systems are built, and will likely yield key insights into neural function.

**Fig. 1:**
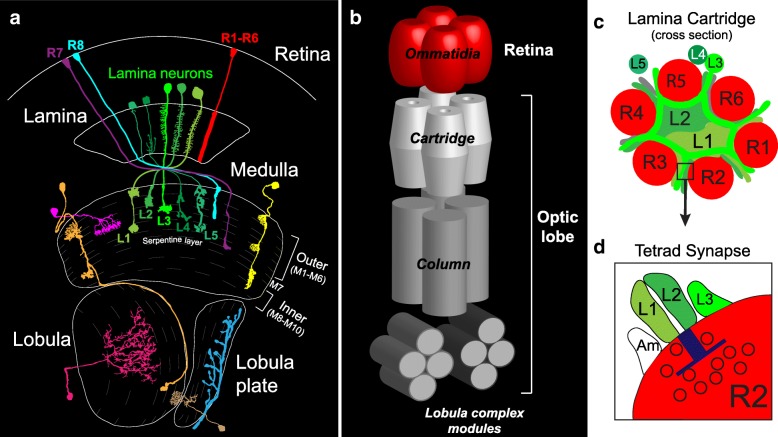
The *Drosophila* visual system. (**a**) Anatomy of the *Drosophila* visual system (Adapted from Fischbach and Diettrich 1989). (**b**) Diagram illustrating the modular organization of the *Drosophila* visual system. Four topographically matched modules from the retina and each region of the optic lobe are shown. Ommatidia (retina), cartridge (lamina), column (medulla), lobula complex modules (lobula and lobula plate). (**c**) Illustration of a cross section through a lamina cartridge. The axons of R1-R6 photoreceptors synapse onto the dendrites of L1-L3 lamina neurons. The R cell axons form a ring around the dendrites, establishing a cylindrical structure that may optimize wiring efficiency. (**d**) R cell axons form tetrad synapses. At each R cell synapse, input is provided to four postsynaptic elements. L1 and L2 are present at every R cell synapse, but the other two components are variable and can include L3, amacrine (Am) or glial (not shown) processes

In the *Drosophila* visual system (see Fig. 1), photoreceptors in the retina detect light and transmit signals to the optic lobe, which comprises four consecutive neuropil regions called the lamina, medulla, lobula and lobula plate. Neurons in the retina and each neuropil region are organized in a modular fashion (Fig. 1b). The retina comprises ~ 750 ommatidial units, each housing photoreceptors (R1-R8) that detect light from specific points in space [1, 2]. Photoreceptors that detect light from the same point in space form connections with the same set of target cells within the lamina (R1-R6) and medulla (R7-R8), forming synaptic modules known as cartridges (lamina) (Fig. 1c) or columns (medulla). Medulla neurons in each column likewise form connections with neurons within modules in the lobula and lobula plate. Thus, the number of modules within each neuropil of the optic lobe matches the number of ommatidia in the retina. And modules across different regions are topographically matched forming columnar circuits that process input from specific points in space. Input from neighboring points in space is processed within neighboring columnar circuits, establishing a retinotopic map in the brain. Within each columnar circuit in the medulla, lobula and lobula plate, visual information is processed by neurons that form connections within specific layers. Thus, in the *Drosophila* visual system columns and layers support two types of parallel processing. Input from different regions of the visual field is processed within parallel columnar circuits, and within each columnar circuit salient visual features are extracted within parallel layers.

This highly stereotyped cellular architecture combined with the ability to study connectivity in a cell type-specific manner at the level of single neurons, makes the *Drosophila* visual system a powerful model for addressing the molecular and cellular bases of columnar and layer organization. Here we will discuss the mechanisms underlying assembly of lamina cartridges and medulla columns, and consider a dynamic model of layer assembly in the medulla implied by recent studies. This article is not meant to be a comprehensive review of the molecules required for column and layer formation in the *Drosophila* visual system. Rather we focus on recent findings to highlight principles governing the assembly of these structures.

## Main text

### Development of the lamina cartridge

To assemble a column, neurons with common properties (e.g. physiological or spatial) converge onto a common set of target cells. This is a complex task because it requires communication between the converging afferents, recognition of the correct target cells and the generation of precise synaptic connections within a complex microenvironment. In the fly visual system, each cartridge in the lamina and column in the medulla contains the same cell types. However, the medulla comprises considerably more cellular complexity. More than 60 cell types innervate the medulla [3, 4] compared to 18 cell types in the lamina [3, 5, 6]. We will first consider the mechanisms underlying assembly of the simpler lamina cartridge.

In the lamina, for the R1-R6 photoreceptors (R cells) and their synaptic targets, the lamina neurons, there is one cell of each type per cartridge, and each cartridge is innervated by R cells that ‘see’ the same point in space [1, 2, 7–9]. Lamina cartridge formation is a choreographed process that appears to be genetically hard-wired. Cartridge assembly begins with R1-R6 cells from each ommatidium extending axons into the lamina as a fascicle [10], with the axons in each fascicle terminating between two layers of glia [11]. Inductive signals from the R cell axons initiate the proliferation and differentiation of lamina neurons in a posterior to anterior fashion that corresponds to the differentiation of R cells in the developing eye disc [12–14]. Since each R1-R6 cell in a single ommatidium receives information from a different point in the visual field, the axons of these cells have to leave the fascicle derived from their ‘home’ ommatidium and integrate with five other R cells from different ommatidia that observed the same point in space. This process occurs between 24 and 40 h after puparium formation (h APF) [15]. How do the R1-R6 cell axons find their appropriate cartridge during this complex developmental process? Evidence from several different studies argue that this is largely dependent on interactions between neighboring R1-R6 growth cones [15–18].

Hiesinger and colleagues used live intravital imaging of R1-R6 growth cones to better understand how R cells find their correct target cartridge. Based on their data they developed algorithms to test whether proper cartridge innervation depends on interactions between R cells and targets in the cartridge, interactions between R cells (afferent/afferent), or both. They found that afferent/afferent interactions were critical for target recognition. Since R cells come into contact with several inappropriate cartridges as they extend, target cues alone were not sufficient to achieve specificity. Only a combination of target + R cell cues produced a model that closely resembled the experimental data, and a model without target cues still worked very well [16]. The algorithm was even able to recapitulate R cell sorting defects that occur at the equator of the retina in wild type flies, providing strong evidence that it accurately represented the biology.

What, then, are the molecular cues that the different R cell subtypes use to recognize each other and targets? Two cadherin proteins, N-cadherin (CadN) and the atypical seven-pass transmembrane cadherin Flamingo (Fmi) have been implicated in this process. CadN is expressed by R1-R6 cells and lamina neurons in each cartridge, whereas Fmi is only expressed by R1-R6 cells [18–20]. Mosaic experiments performed by Clandinin and colleagues have shown that CadN is cell autonomously required in R1-R6 cells and non-autonomously required in lamina neurons for proper cartridge innervation. Interestingly, CadN was found to be required in all lamina neuron subtypes (L1-L5), even those that are not postsynaptic to R1-R6 cells (i.e. L4 and L5). Thus, while CadN-based R cell-lamina neuron interactions are necessary for correct innervation of the target cartridge, additional mechanisms are likely to regulate synaptic specificity between these cells [21].

These experiments argue that molecular cues in the target region are necessary for R cell sorting, in contrast to the model proposed by Langen et al., and suggest that the developmental algorithms may require further refinement. A simple way to address these differences would be to perform the live imaging experiments on animals that lack CadN in lamina neurons. If the developmental algorithm is correct, R cells should find their cartridges correctly even when lamina neurons lack CadN.

In contrast to the target-derived cue data, the genetic and the modeling experiments related to afferent-afferent interactions are consistent with one another. Fmi appears to be the primary molecular cue on afferents that mediates R cell interactions. Interestingly, Fmi is strictly non-autonomously required in R cells for cartridge innervation. Clones of *fmi* mutant R1-R6 cells target normally, but their neighboring wild-type cells do not [20]. Given that the level of Fmi protein expression is different between R1-R6 cells, it was proposed that these differences provide instructional information for targeting. Since an *fmi* mutant R cell would not be able to detect these differences on either side of its growth cone, it would not be affected. Wild-type R cells, however with Fmi interactions on one, but not the other side of their growth cone would mis-target due to this imbalance. Other cues, however, are likely to work in concert with Fmi since gross mis-targeting is observed when most of the R1-R6 cells lack Fmi [20]. In summary, assembling R cells into the cartridges in the lamina is genetically programmed and requires communication between neighboring R cell growth cones. Merging live-imaging and genetic techniques should provide a way to address the more controversial role of afferent-target interactions during the cartridge selection process.

### Synapse formation within the lamina cartridge

Within each cartridge the neurites of R cells and lamina neurons organize into a stereotyped arrangement that is thought to optimize the placement of axons and dendritic processes for efficient synapse formation. In lamina cartridges, R cell axons synapse with the dendrites of lamina neurons L1-L3, while L4 and L5 do not receive R cell input. L1 and L2 participate at every R cell synapse, and L3 is present at a subset of these [5, 6]. A cross section through a cartridge (Fig. 1c) shows that the six R cell axons form a circle around a central core containing L1 and L2 neurites, which extend dendrites midway through pupal development to form synapses with R cell terminals. The main neurites of L3, L4 and L5 are located in the periphery of each cartridge (L3 projects dendrites into the cartridge core during mid pupal development). Interestingly, this stereotypic cartridge organization depends on differential adhesion mediated by CadN [22]. L1 and L2 express high levels of CadN, whereas R cells and the other three lamina neurons that are located on the cartridge periphery, express lower levels of CadN. Manipulations that alter this relationship, such as removing CadN from lamina neurons or overexpressing it in R cells, displace L1 and L2 from the center to the periphery of the cartridge. Thus, it appears that the strength of CadN adhesive interactions determines whether neurites are located at the cartridge core or periphery. This organizational strategy likely places L1 and L2 in a position that is optimal for participating in every R cell synapse.

**Fig. 2:**
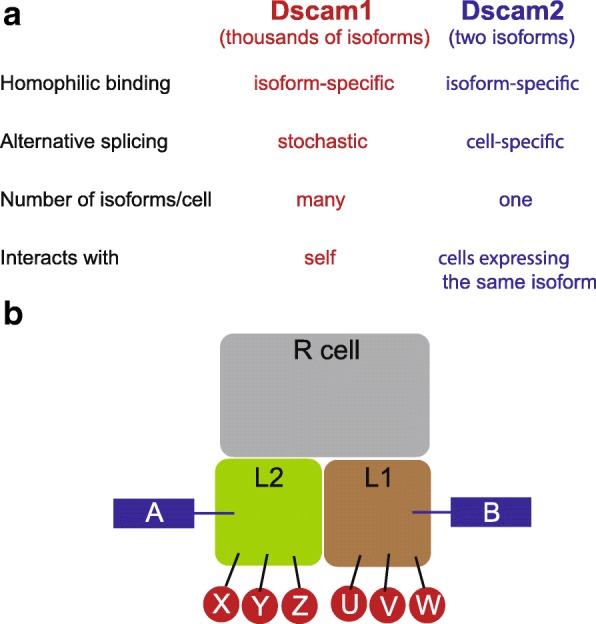
Alternative splicing of Dscam1 and Dscam2 regulates synaptic exclusion. (**a**) Properties of Dscam1 and Dscam2 alternative splicing are very different, but allow both to exclude processes from the same cell at tetrad synapses. (**b**) A schematic of a tetrad synapse (variable components not shown). A random array of Dscam1 isoforms are expressed in L1 and L2. Since these isoforms are not identical between the two cells, homophilic repulsion does not occur. L1 and L2 express distinct isoforms of Dscam2. This allows for self-repulsion, but not repulsion between the two different cells. Through this indirect mechanism of excluding inappropriate partners at synapses, postsynaptic specificity is achieved

Synapses form in an *en passant* fashion along R cell axons with ~ 50 synapses forming per axon terminal (300 per cartridge) [5, 6]. R1-R6 cells form tetrad synapses that include four distinct postsynaptic elements (Fig. 1d). Invariantly, one dendritic process from an L1 lamina neuron is paired with an L2 process at every synapse, and the other two components are variable, and can include L3, amacrine and glial processes [5, 6]. L1 and L2 neurons represent distinct arms of the motion detection circuitry [23, 24], and providing equal input to these cells may be important for motion vision. Pairing L1 and L2 dendrites at each synapse is challenging as there are thousands of dendritic branches produced by L1 and L2 within the confined space (~ 5µm × 30µm) of the cartridge. L1-L2 pairing is achieved through a process called synaptic exclusion [25], that involves repulsion between processes of the same cell. Synaptic exclusion prevents postsynaptic pairings of two elements from the same cell (e.g. L1-L1 or L2-L2) at a synapse. Dscam1 and Dscam2, two transmembrane immunoglobulin superfamily proteins, are expressed in L1 and L2 and function redundantly to regulate synaptic exclusion by mediating self-avoidance [26] (see Fig. 2). Synaptic exclusion relies on alternative splicing of these two genes. Alternative splicing within the extracellular domains of both *Dscam1* and *Dscam2* results in isoform-specific homophilic binding proteins, and homophilic binding induces repulsion [27–31]. Alternative splicing of the over 38,000 *Dscam1* isoforms is stochastic, many isoforms are expressed in each neuron and only neurons with identical isoforms can mediate homophilic binding that leads to repulsion. Thus, individual neurons appear to have a unique ‘Dscam1 identity’ that only permits self-interactions [32]. Removing *Dscam1* from lamina neurons, however, does not significantly disrupt synaptic exclusion. Synapses that contain multiple elements from L1 or L2 are only observed when *Dscam1* and *2* are simultaneously disrupted. Under these conditions there is a randomization of L1 and L2 at each synapse [26]. *Dscam2* alternative splicing, in contrast to *Dscam1*, is regulated in a cell type-specific manner. The *Dscam2* gene encodes two different extracellular isoforms (A and B) and most cell types express either Dscam2A or Dscam2B, not both. For example, L1 cells express isoform B and L2 cells express isoform A [33]. Since A cannot bind with B, it was hypothesized that this would allow Dscam2 to mediate repulsion between branches of the same L1 or L2 cell, but not between L1 and L2 processes within the same cartridge. If L1 and L2 expressed the same Dscam2 isoform, one would expect inappropriate repulsion between these cells and perturbed synapses. Recent studies have confirmed that this is the case. In animals expressing a single Dscam2 isoform, there is a reduction in photoreceptor synapses and a reduction in the complexity of L1 and L2 dendrites, consistent with inappropriate repulsion between these cells when they express identical Dscam2 isoforms [34]. Thus, the mechanism for forming the postsynaptic L1-L2 pair at every R1-R6 synapse is indirect. Pairing of two elements from the same cell is prevented through synaptic exclusion, which involves repulsion between branches of the same cells and is driven by stochastic alternative splicing of *Dscam1* and regulated alternative splicing of *Dscam2.*

Although these studies address how proper L1-L2 pairing is regulated at each synapse, it’s still unclear how R cells form synapses with the appropriate targets. For example, how the variable postsynaptic components of the tetrad are specified is unknown, and raises complications to the synaptic exclusion model. L3 cells express the same isoform of Dscam2 as L2 cells, yet each synapse containing an L3 process also contains a process from L2. How repulsion between these processes is prevented has not been addressed. In addition, molecules that mediate the specificity of R cells for L1-L3, but not L4 and L5 neurons have not been identified, and whether adhesive interactions between postsynaptic components within each tetrad are important for synapse formation is not known. Thus, many questions about how these relatively simple synaptic modules get wired up, still remain.

### Columnar restriction in medulla columns

In contrast to lamina cartridges, which receive input from identical afferents that synapse onto the same targets, each medulla column is innervated by different types of afferent neurons that synapse with different types of target cells. In addition, many more cell types form connections in medulla columns compared with cartridges in the lamina. Here we will focus on the mechanisms underlying column formation in the medulla.

**Fig. 3:**
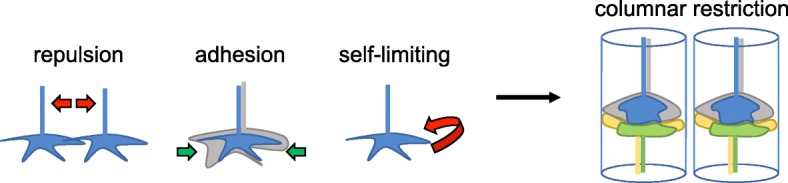
Multiple mechanisms for restricting processes to single columns. Columnar restriction can be achieved through repulsion between neighboring cells of the same type, adhesion to cells within the same column and autocrine signaling that limits growth cone movement. The end result is that connections are made within the column rather than with correct target cells that reside in neighboring columns

The medulla (Fig. 1) receives input directly from color photoreceptors R7 and R8, which are tuned to UV or blue/green light, respectively, and indirectly from broadly tuned R1-R6 cells through lamina neurons L1-L3, which function in motion detection [23, 24]. Within each column, R7, R8 and lamina neurons carrying input from the same point in space innervate the same column and synapse with specific types of medulla interneurons and projection neurons (e.g. Mi and Tm) that process and relay information to the lobula and lobula plate. The medulla comprises cells that only innervate single columns (uni-columnar), and cell types that integrate information from multiple columns (multi-columnar). This discussion will concentrate on how the neurites of uni-columnar cells are restricted to single columns (see Fig. 3).

Tiling is one way in which neurites are restricted to a single column. The term ‘tiling’ was originally coined by Boycott and colleagues when describing the dendritic fields of neurons in the cat retina. The dendrites of neighboring cells extended until they encountered processes from another cell of the same type [35]. The mosaic of non-overlapping cells looked like tiles on a floor and were proposed to be generated through cell-type-specific repulsive cues. Visualizing the ~ 750 medulla columns in cross-section through a synaptic layer reveals a similar arrangement. Cells of the same type arborize at a specific layer in their “home” columns, but do not extend into neighboring columns (several microns away), even though these also contain appropriate synaptic targets. L1 neurons, whose axons arborize in two distinct synaptic layers within the medulla, use Dscam2 repulsion to restrict their processes to a single column. During development, L1 processes from neighboring columns overlap as they search for their postsynaptic targets. Dscam2 isoform B is expressed on the surface of these overlapping processes and induces contact-dependent repulsion between them, causing retraction of the extended processes thereby restricting innervation to the home column [27]. In contrast, L2 cells, which express isoform A of Dscam2, are able to tile the medulla independent of Dscam2. This demonstrates that there must be multiple mechanisms for preventing overlap between cells of the same type in neighboring columns.

Selective adhesion to neurons within the correct column has also been proposed to mediate columnar restriction. CadN, which functions at multiple levels of column organization, plays a role in restricting the processes of L5 lamina neurons to a single column. When *CadN* is removed from L5 specifically, the terminal arbor of L5 invades neighboring columns. This tiling phenotype of the terminal branch is autonomous to L5 and presumably due to interactions with other CadN expressing neurons in this layer [36], such as L1.

A third mechanism, involving autocrine or paracrine activation of the TGF-β signaling pathway has been found to regulate the columnar restriction of axonal and dendritic processes, respectively [37, 38]. To illustrate this mechanism we will focus on the autocrine pathway that limits R7 axons to a single column. Mutations in *Importinα3* (*imp-α3*) and *Baboon* (*babo*), were found to cause defects in a UV-visible light preference test that requires the function of R7 photoreceptors [37]. Imp-α3 is a nuclear import factor and babo is a type I TGF-β/Activin receptor. Analysis of R7 clones mutant for either gene revealed that axons correctly innervated the target layer but frequently sent processes into neighboring columns, thus exhibiting a tiling phenotype. By generating mutant clones in a background where neighboring R7 cells were missing, it was found that the penetrance of the tiling phenotype increased significantly, providing evidence that another partially redundant pathway exists. It was proposed that this redundant pathway consisted of a repulsive signal, whereas the TGF-β pathway works through transcription-dependent factors that regulate growth cone motility or synapse formation [37]. Consistent with this hypothesis, Rao and colleagues identified the immunoglobulin superfamily transmembrane protein, Turtle (Tutl) as a tiling receptor in R7 neurons [39], which is thought to function in a manner similar to Dscam2.

Collectively, these studies demonstrate that cellular complexity influences the mechanisms utilized to assemble columnar units. In the lamina, afferent/afferent and afferent/target interactions mediated by a few broadly expressed cell adhesion molecules are sufficient for columnar specificity. And within cartridges synapse formation is regulated by a process of synaptic exclusion mediated by broadly expressed homophilic cell surface molecules that undergo alternative splicing in a stochastic or regulated manner (Fig. 2). While in the medulla, which is more complex, diverse mechanisms, including repulsion, adhesion and autocrine regulation of growth cone dynamics function in a cell-specific manner to restrict neurites to single columns (Fig. 3). How most cell types in the medulla achieve columnar restriction is still unclear, thus it is likely that additional mechanisms remain to be identified. In addition, some neurons, such as L3 lamina neurons, have processes that innervate neighboring columns but primarily form synapses within the home column. In these contexts, synapses but not neurites are restricted to columns. How this is regulated is unknown.

### Tiling in vertebrates

Columns are present in many regions of the vertebrate cortex, but how cells restrict their processes to a single unit has not been described. A recent study on the role of clustered protocadherins in the development of serotonergic neurons in the mouse, however, provides a strong indication that mechanisms similar to what have been previously observed in flies regulate neurite spacing in higher vertebrates.

Clustered protocadherins (Pcdh) are isoform-specific homophilic binding proteins that appear to perform similar functions to Dscam proteins in flies. There are three *protocadherin* gene clusters that through alternative promoter selection can generate over 50 different isoforms. These proteins form complexes *in cis* and only identical protocadherin complexes on opposing membranes can mediate homophilic binding [40]. The *protocadherin-γ* gene cluster was shown to mediate self-avoidance in starburst amacrine cells [41], through the stochastic expression of many isoforms in each cell [42–44], a role reminiscent of fly *Dscam1*. By contrast, serotonergic neurons express a subset of Pcdh isoforms [42], and in mutants that lacked cytoplasmic exons common to all *Pcdhα* isoforms, serotonergic neurons exhibited defective projections [45]. More directed genetic analyses by Maniatis and colleagues revealed that a single isoform, Pcdhαc2, was autonomously required in these cells and that it functions as a tiling receptor between neighboring serotonergic neurons [46]. Serotonergic neurons exhibited extensive reorganization, overlap between neighboring serotonergic neurons and clumping in conditional alleles that removed *Pcdhαc2*; the mice also exhibited depressive behaviors [46]. Thus, *Pcdhαc2* appears to function similar to *Dscam2* in the medulla of the fly, mediating repulsive interactions between cells of the same type.

### Do columns contribute to brain function?

An outstanding question in the field is whether columnar organization is required for brain function. This is difficult to address using classic genetic approaches because mutations that disrupt columns often result in other wiring defects. Natural differences in the cortical columns of some vertebrates, however, could provide a means for addressing this question.

Vertebrate columns have largely been defined based either on receptive fields using electrophysiology, or by their expression of different metabolic enzymes, such as cytochrome oxidase. Unlike *Drosophila*, the cellular makeup and development of these modules has not been well-described. Many columns have been identified that represent distinct sensory modalities, including motor, auditory and visual stimuli [47]. Ocular dominance columns have been particularly well-studied in many different species. Axons from the lateral geniculate nucleus assemble into alternating columns from either the left or right eye in the visual cortex of numerous animals [48], including humans. It has been hypothesized, although not yet demonstrated, that segregating inputs from different eyes plays a role in binocular vision [49]. However, some animals, like the tree shrew, completely lack ocular dominance columns [50] and others, like the squirrel monkey, show considerable variation in column formation between animals and even within individual animals [51]. Based on these observations it has been suggested that ocular dominance columns serve no function in terms of vision [47, 52]. The rationale is that if these columns did serve an important function, they would have been maintained through natural selection. There are many other explanations, however, and although the evolutionary argument is a strong one, it needs to be verified experimentally. While the functional relevance of columnar organization remains unclear, at the very least it can simplify brain development and decrease the error rate of neuron targeting by compartmentalizing neurons with similar properties.

### Layers within columnar structures

In parallel to the mechanisms described above that organize the regular spacing of cells horizontally, additional cues regulate spacing in the vertical direction often forming refined synaptic layers. In general, different layers comprise different complements of cell types, and the arborizations of different types of input cells are confined to specific layers. This provides a structural basis for processing different information in parallel. Within the *Drosophila* visual system layers are particularly well-defined in the medulla, where input from different classes of photoreceptors converges. Over the past decade, developmental studies have begun to illuminate how specific medulla layers develop. They imply that layers are not pre-defined but form dynamically from broad domains. Here, we will describe the layered architecture of the medulla, and discuss key findings that support a dynamic model of layer assembly. To gain a more comprehensive view of the molecules that regulate circuit formation within the medulla see [53–55].

### The medulla is a layered synaptic network

The medulla (see Fig. 1a) comprises ten layers M1-M10, which are divided into outer (M1-M6) and inner (M8-M10) regions by tangentially projecting processes that form the serpentine layer (M7). The cell bodies of medulla neurons are located in the medulla cortex, which surrounds the layered neuropil region. Medulla layers are defined by the morphologies of the axons and dendrites of specific cell types. Using the Golgi impregnation method Fischbach and Dittrich identified more than sixty cell types that innervate the medulla in adult flies [3] (more recent studies indicate this number is even larger [4]). They discovered that the neurites of single neurons of the same type, as determined by their identical morphologies, occupied a characteristic depth within the neuropil, and frequently found that the neurites of different cell types either overlapped completely or occupied mutually exclusive positions. Using this criteria, they defined ten parallel layers. Serial section electron microscopy (EM) analyses have revealed that the positions of terminals and branches are largely predictive of where synapses form [56–58]. Although some neurons do not have obvious terminals and form synapses *en passant*.

Functional studies indicate that the layered organization of the medulla reflects functional differences between neurons. The presentation of motion stimuli was found to elicit high levels of glucose uptake within specific layers in a stimulus-specific manner [59]. These studies also revealed consistent coupling of glucose uptake between specific outer and inner medulla layers, suggestive of preferential connectivity between neurons within these layers and the existence of physiological layer-specific circuits. More recently, genetic silencing experiments have provided evidence that lamina neurons L1 and L2, which arborize within different medulla layers, provide input to functionally distinct motion detection circuits [60, 61].

Collectively, these morphological and functional studies demonstrate that the medulla comprises a highly ordered, layered synaptic network, and that this organization reflects functionally distinct pathways.

### Targeting to outer or inner medulla regions

Different classes of neurons form connections within the outer medulla, inner medulla or both regions in a characteristic manner. Studies investigating the targeting of lamina neurons and medulla intrinsic neurons have begun to shed light on the mechanisms that regulate targeting to the outer or inner medulla.

**Fig. 4:**
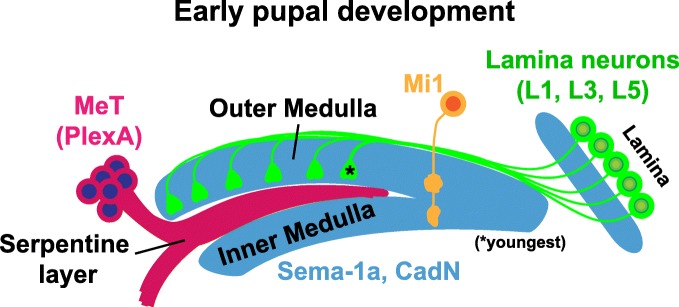
Targeting to the outer or inner medulla. A diagram of medulla development at an early pupal stage (~12 hours after puparium formation [h APF]). Lamina growth cones expressing CadN and Sema-1a are prevented from innervating the inner medulla through repulsive interactions with PlexA expressing medulla tangential cells (MeT), and interactions with other CadN expressing processes in the outer medulla. Mi1 = a medulla instrinsic 1 neuron. The asterisk indicates the youngest lamina neuron axons within the medulla neuropil

Lamina neurons exclusively innervate layers within the outer medulla (Fig. 1a). At an early stage of pupal development, the growth cones of lamina neurons L1, L3 and L5 terminate in a proximal domain within the outer medulla near the developing serpentine layer (see Fig. 4). These neurons are prevented from targeting more proximally, into the inner medulla, through a common mechanism [62]. This involves adhesion within the proximal domain of the outer medulla, mediated by CadN, and repulsion from the sub-adjacent processes of medulla tangential cells (MeT) within the serpentine layer, mediated by Semaphorin-1a (Sema-1a)/PlexinA (PlexA) interactions. Disrupting either *CadN* or *Sema-1a* in L1, L3 or L5 neurons caused a small subset of their axons to mis-target beyond the outer medulla. However, disrupting both genes simultaneously in each cell type caused a large fraction of the growth cones to mis-target to the serpentine layer and inner medulla, indicating that CadN and Sema-1a function synergistically in this context.

It was proposed that the functions of CadN, Sema-1a and PlexA are established by their complementary patterns of expression [62] (Fig. 4). CadN and Sema-1a are enriched on lamina growth cones and other neurites within the outer and inner medulla, and weakly expressed in the serpentine layer. Conversely, PlexA is predominantly expressed on neurites within the serpentine layer (e.g. MeT neurites) and is weakly expressed in the outer and inner medulla. L1, L3 and L5 axons and MeT neurites project into the medulla in a coincident manner. Lamina axons project into the outer medulla perpendicular to developing layers, and MeT neurites innervate the developing serpentine layer orthogonal to lamina axons (Fig. 4). It was proposed that when the processes meet at the outer medulla/serpentine layer border (Fig. 4, asterisk), repulsive Sema-1a/PlexA interactions act as a barrier to the lamina axons. At the same time, CadN-mediated adhesion between lamina axons, between lamina axons and other processes in the outer medulla, or both, equally prevents axon growth. It was further suggested that the timing of MeT innervation might allow earlier targeting sema-1a expressing medulla neurons (potentially Mi neurons) to innervate the inner medulla, wherein Sema-1a may be necessary for patterning connections.

Hasegawa and colleagues demonstrated that CadN also plays a role in targeting axons to the inner medulla [63]. Medulla intrinsic 1 neurons (Mi1) elaborate dendrites in outer layers M1 and M5, and target axons that innervate the M9 and M10 inner medulla layers. By the end of larval development (0 h after puparium formation [h APF]) Mi1 neurons have formed arborizations in the outer and inner medulla roughly corresponding to positions in M5 and M9/10, respectively (Fig. 4) (refined layers are not evident at this early stage of medulla development). The distal M1 arborization forms later in development. When *CadN* is disrupted in Mi1 neurons, a significant fraction of the neurons fail to innervate the inner medulla at 0 h APF, but still arborize at a depth consistent with the developing M5 layer in the outer medulla. Thus, in this context, CadN is dispensable for innervation of the outer medulla but necessary for targeting to the inner medulla. The CadN expressing targets of Mi1 neurons in the inner medulla were not identified, and it remains unknown whether Sema-1a also regulates Mi1 targeting.

Collectively, these findings show that the outer medulla, inner medulla and the serpentine layer that divides them are defined by the complementary expression of CadN, Sema-1a and PlexA. CadN and Sema-1a act in an overlapping manner at the boundary of the outer medulla and serpentine layer to restrict specific lamina axons to the outer medulla [62]. By contrast, in a subclass of medulla intrinsic neurons that innervate both outer and inner regions, CadN function is necessary for innervation of the inner medulla, but dispensable for arborization in the outer medulla [63]. These studies indicate that broadly expressed adhesive and repulsive molecules act in a context dependent manner to regulate targeting to general outer and inner medulla regions, and suggest that the timing of medulla innervation may influence whether processes innervate the outer medulla only, or both outer and inner regions.

### Development of discrete layers

Outer medulla layers (M1-M6) are primarily defined by the morphologies of lamina neuron and photoreceptor (R7, R8) axons in adult flies [3]. Studies investigating the development of these axons have illuminated mechanisms that give rise to discrete layers.

#### Innervation of broad domains

**Fig. 5:**
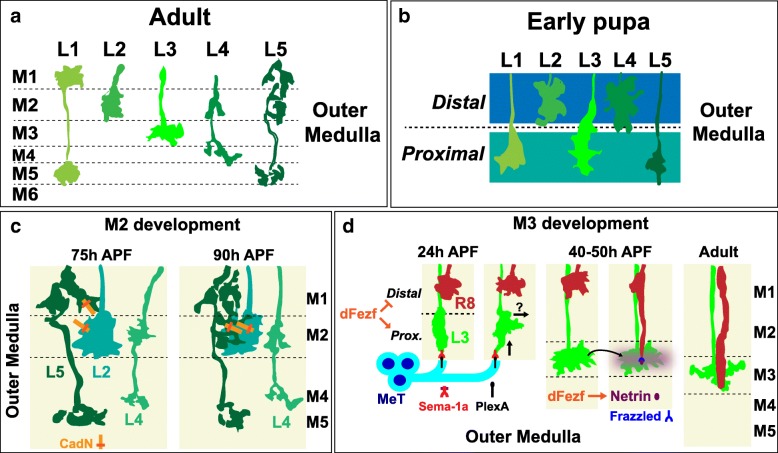
Outer layers develop in a stepwise manner from broad domains. h APF = hours after puparium formation (**a**) A representation of the adult morphologies of lamina neuron axons L1-L5. The arborizations of lamina neuron axons help define specific outer medulla layers. (**b**) A drawing of lamina neuron growth cones L1-L5 in early pupal development. Prior to arborizing in discrete layers lamina growth cones terminate in distal or proximal domains within the outer medulla. (**c**) An illustration of M2 development. A CadN-dependent interaction between the axons of lamina neurons L2 and L5 mediates the branching of L5 axons into the M2 layer. (**d**) A diagram of M3 development. The M3 layer develops in part through the sequential innervation of L3 and R8 axons. DFezf cell autonomously promotes the targeting of L3 growth cones to the proximal domain of the outer medulla. L3 growth cones then segregate into the developing M3 layer in part through repulsion from medulla tangential fibers (MeT). DFezf activates the expression of *Netrin* which is secreted from L3 growth cones, and serves as an M3-specific cue for R8 growth cones. (Arrows in the second panel from the left indicate the retraction of the leading edge of an L3 growth cone, and extension of filopodia laterally across the column within the developing M3 layer. The arrow in the third and fourth panels from the left show the secretion of Netrin from L3 growth cones, which becomes concentrated within the developing M3 layer)

Fischbach and Dittrich used the nearly mutually exclusive positions of L1-L5 arborizations to help define layers M1-M5 [3] (Fig. 5a). However, while lamina axon arborizations define five discrete layers in adult flies, in early pupal development lamina neuron growth cones terminate in two broad domains within the outer medulla [36, 62] (Fig. 5b). The growth cones of L2 and L4 neurons terminate in a distal domain, and L1, L3 and L5 growth cones terminate in a proximal domain. This suggests that, early in medulla development, discrete outer layers are not well defined, and that layers are refined overtime from initially broad regions.

A recent study from my laboratory has begun to shed light on the mechanisms underlying broad domain specificity within the early outer medulla. We found that *Drosophila* Fezf (dFezf), an evolutionarily conserved transcription factor that is exclusively expressed in L3 neurons in the lamina, is necessary and sufficient for targeting to the proximal domain of the outer medulla [64]. Disrupting dFezf in L3 neurons caused incorrect growth cone termination in the distal domain of the outer medulla in early pupal development, and innervation of layers distal to the L3 target layer M3 (i.e. M1/M2) in adult flies. Mis-expression of dFezf in L2 neurons caused their growth cones to inappropriately terminate in the proximal domain of the outer medulla in early pupal development, and innervate the M3 layer in adult flies (WT L2 neurons exclusively innervate M2). Taken together, these studies show that dFezf promotes targeting to the proximal domain of the outer medulla and innervation of the M3 layer, and indicate that broad domain specificity in early pupal development is essential for proper layer specificity in adult flies.

We also demonstrated that dFezf regulates L3 growth cone targeting in parallel to CadN and Sema-1a, and that *dpr* genes, which encode a family of cell surface proteins [65], are prominent direct or indirect dFezf targets. Dprs are immunoglobulin domain (Ig) containing proteins that bind heterophilically to other Ig proteins called dpr-interacting proteins (DIPs) [66, 67]. Lamina neurons differentially express dprs, and their synaptic targets in the medulla express matching DIPs [68]. In *dFezf* null L3 neurons *dprs* normally expressed in L3 were downregulated, and *dprs* expressed by other lamina neurons (especially L2 and L4) were upregulated [64]. Based on this we propose that dFezf regulates growth cone targeting by activating the expression of dprs that mediate interactions with target cells in the proximal domain of the outer medulla, and by repressing the expression of dprs that mediate interactions with targets in the distal domain. In this model CadN and Sema-1a function in parallel to dpr-DIP interactions to consolidate growth cone position within the proximal domain. However, as many genes encoding cell surface proteins display altered expression in *dFezf* null L3 neurons in addition to *dprs*, the mechanism by which dFezf controls broad domain specificity remains to be determined.

#### Refinement of discrete layers within broad domains

During the course of pupal development, the medulla expands as neurons branch and form arborizations, and later born cells project neurites into the neuropil [36, 62, 69]. During this time, specific lamina neuron and photoreceptor axons undergo local rearrangements or form additional arbors, which contribute to the development of layers M1-M5. Developmental studies have provided insight into some of the mechanisms governing formation of M2 and M3. Here we will focus on these layers, as much less is known about the development of other layers.

#### M2

The M2 layer contains the entire L2 arborization and also processes from L4 and L5 (Fig. 5c). Within M2, L5 and L2 neurons form reciprocal connections but do not synapse with L4 neurons [57, 58]. In early pupal development, L2 axons arborize within the distal domain of the outer medulla [36, 62]. This domain develops into the M2 layer, in part through the branching of L5 axons late in pupal development [36] (Fig. 5c). The distal L5 arborization begins to form in the M1 layer during mid-pupal development (~ 48 h APF), and branches into the M2 layer between 75 and 90 h APF. L5 branching into the M2 layer is mediated by an CadN-dependent interaction between L2 and L5 axons [36]. CadN is localized to the arborizations of both neurons during pupal development [36, 62], and is required cell autonomously in L5 neurons and non-autonomously in L2 neurons for the proper branching of L5 axons. Disrupting *CadN* in L5 neurons inhibits branching into the M2 layer, while arborization in M1 still occurs. In addition, disrupting *CadN* in single L2 neurons causes wild type L5 neurons in the same columns to preferentially branch into neighboring columns, presumably through CadN-mediated interactions with wild type L2 neurons. Thus, in this context, CadN-dependent interactions regulate both layer specificity and columnar restriction. Since CadN is also expressed by L4 neurons, synaptic specificity between L2 and L5 is likely driven by other cues. Nevertheless, this shows that adhesive cell-cell interactions between synaptic partners are important for layer innervation.

#### M3

Studies of M3 development show that interactions between non-synaptic partners are also important for layer formation, and that specific mechanisms are dedicated to coordinating the layer innervation of different cell types. The M3 layer receives input from L3 lamina neurons and R8 photoreceptors. Within each medulla column, L3 and R8 axons that carry input from the same point in visual space terminate in the M3 layer and synapse with shared and unique targets, but not with each other [57, 58]. L3 axon terminals stratify exclusively within M3. R8 axons form *en passant* synapses in multiple layers but terminate in the M3 layer. L3 and R8 axons innervate the M3 layer sequentially during pupal development (Fig. 5D). R8 neurons are born before L3 neurons [70] and project axons that initially terminate near the superficial (distal) surface of the medulla [69, 71, 72], where they remain for up to two days. L3 axons project past R8 axons and terminate in a domain within the proximal outer medulla shared with the growth cones of L1 and L5 neurons [36, 62] (~ 24 h APF) (discussed above). At this stage L3 growth cones are broad, spanning most of the outer medulla. Subsequently, L3 growth cones segregate away from the proximal domain of the outer medulla to a more distal position by undergoing a stereotyped growth cone rearrangement [62]. This involves retraction of the leading edge, which is partially regulated by Sema-1a/PlexA repulsion from processes in the serpentine layer, and extension of the growth cone laterally across the column within the nascent target layer, which occurs through an unknown mechanism. As a result of this process L3 growth cones are reshaped into globular structures confined to the developing M3 layer (~ 40 h APF). In addition, as the M5 layer is defined by L1 and L5 terminals (Fig. 5a), and L1 and L5 growth cones maintain their positions within the proximal domain of the outer medulla (Fig. 5b), the departure of L3 growth cones from this region also contributes to M5 development.

Within developing M3, L3 growth cones secrete Netrin, which becomes concentrated within the layer [73, 74]. Coincidently R8 growth cones extend from the medulla surface to the M3 layer wherein interaction between Netrin and its receptor Frazzled, localized on R8 growth cones, is necessary for R8 layer specificity [73]. Disruption of *Netrin* or *Frazzled* caused R8 growth cones to inappropriately terminate at superficial positions (e.g. M0-M2) [73]. In vivo time-lapse imaging showed that when Netrin/Frazzled signaling is blocked R8 growth cones extend and target to the M3 layer normally, but are unable to maintain position in the layer and retract [75]. Based on this it was concluded that the Netrin/Frazzled pathway regulates the attachment of R8 growth cones within the M3 layer, and that other mechanisms regulate R8 extension and “recognition” of M3. The cell surface molecules Flamingo and Golden Goal have been proposed to function in the same pathway to regulate the targeting of R8 axons from M0 to M3 and may act in parallel to the Netrin/Frazzled pathway to control this step (see [55, 76]). Interestingly, we found that *Netrin* expression in L3 neurons is activated by dFezf [64]. Disrupting dFezf in L3 abolished Netrin protein expression within the M3 layer and caused defects in R8 layer specificity reminiscent of those induced by a *Netrin* deletion. Thus, in addition to cell autonomously instructing broad domain and layer specificity in L3 neurons, dFezf non-autonomously regulates R8 layer specificity through activation of a secreted molecule (Netrin).

To summarize, the M3 layer develops in part through the sequential innervation of L3 and R8 axons (Fig. 5d), and R8 layer specificity relies on a signal (Netrin) from L3 neurons. As L3 and R8 do not form synaptic connections, this demonstrates that interactions between non-synaptic partners are important for layer formation. In addition, the M3-specific innervation of both L3 and R8 is coordinated by dFezf. DFezf functions cell autonomously to promote L3 layer specificity, potentially by regulating a program of *dpr* expression, and non-autonomously to regulate R8 layer specificity via activation of *Netrin*. This suggests that the stepwise assembly of specific layers is regulated by transcriptional modules that cell-intrinsically target neurons to the correct layer, and cell-extrinsically recruit other circuit components (see below).

While significant progress has been made in understanding how the M2 and M3 layers form, we are really just scratching the surface. Dozens of cell types form connections within each medulla layer, and for a given layer the temporal order of innervation of different neuron types is unknown, as are the underlying molecular and cellular mechanisms except in a few instances (some of which are described above). Given the considerable complexity of cellular processes that make up specific medulla layers, it is likely that diverse mechanisms contribute to the development of each layer.

#### Synaptic specificity within layers

Once within layers, how do neurons distinguish between appropriate and inappropriate synaptic partners? One possibility is that neurites simply synapse onto targets in close proximity within the target layer. However, several lines of evidence from EM studies argue that, at least to some degree, molecular determinants regulate synaptic specificity. First, the degree of contact between processes is not always predictive of synaptic connectivity. For example, L3 and R8 axons contact each other extensively within the medulla, but do not form synapses [56–58]. Second, within each medulla column each neuron forms most of its synapses with a specific set of cell types, and this set of synaptic partners remains consistent between different columns [57, 58]. And finally, when a specific neuron (Mi15) was found to be missing from a column (home column), it was discovered that downstream targets within the home column extended neurites into neighboring columns and synapsed with Mi15 cells within these columns, rather than forming connections with alternative partners in the home column [58]. Thus, within layers molecular mechanisms are likely to regulate how neurons discriminate between correct and incorrect synaptic partners.

A recent study has suggested that the differential expression of members of specific cell surface families may encode synaptic specificity. Tan and colleagues found that, during pupal development, dpr and DIP Ig proteins are expressed in a complementary manner between afferents (i.e. lamina neurons, R7, R8) and their medulla neuron targets [68]. R7, R8 and each lamina neuron subclass express multiple dprs in unique combinations, and subsets of their synaptic targets express matching DIPs. Based on these patterns of expression, it was proposed that different heterophilic dpr-DIP interactions, or combinations of them, encode synaptic specificity in these neurons [68]. Interestingly, dpr expression was found to be dynamic during pupal development. While some dprs were expressed in the same cells throughout, other dprs were only expressed in early or late stages, and some became expressed in different cell types at different stages. This suggests that dpr-DIP interactions may regulate early and late steps of circuit formation in a context-dependent manner. Carrillo and colleagues showed that a specific dpr-DIP interaction between R7 photoreceptors and their primary synaptic targets, distal medulla 8 neurons (Dm8), is necessary for Dm8 survival [66, 67]. However, whether cell death resulted from deficits in synaptic connectivity or a lack of trophic support is unclear. Thus, while dpr and DIP proteins are good candidates for regulating synaptic specificity, how they function remains to be determined.

In addition to genetic mechanisms, activity may play a role in shaping connections between neurons in layers. After innervating their target layers, some neurites undergo a process of refinement that leads to their characteristic morphologies [36, 62, 64, 69]. For example, within the developing M3 layer, globular L3 growth cones transform into flattened terminals that stratify within the proximal region of M3. Coincidentally, the dendritic processes of transmedullary 9 neurons (Tm9), which receive input from L3, also become refined into thin branches within the M3 layer. While it is unclear whether refinement plays a role in synaptic partner selection and how it is regulated, it’s possible that it is driven by synaptic activity. Additionally, while EM studies show that neurons form connections with a common set of synaptic partners in each column, the numbers of synapses formed between the same neurons in different columns can vary considerably [58]. Thus, synaptic activity may also regulate the strength of particular synaptic connections. Visualizing and manipulating neural activity in a cell type-specific manner during development and in adult flies will provide a way of determining the degree to which genetic and activity-dependent mechanisms interact to specify neural connectivity.

### A dynamic model of layer assembly in the medulla

**Fig. 6:**
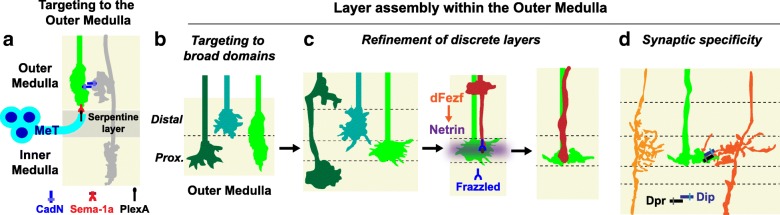
A dynamic model of layer assembly in the medulla. Outer medulla layers are established in a stepwise manner during development through a precise sequence of interactions between specific cell types. To illustrate this, the figure concentrates on the stepwise targeting of L3 lamina neuron axons within the medulla during pupal development. (**a**) L3 axons (green) are prevented from innervating the serpentine layer and inner medulla by adhesive (CadN-dependent) and repulsive (Sema-1a/PlexA) interactions, that serve as a barrier to further extension. MeT = medulla tangential neurons. The gray neuron represents a potential CadN expressing target of L3 axons. (**b**) Prior to innervating the target layer, L3 axons (light green) terminate in a proximal domain of the outer medulla shared by the growth cone of another lamina neuron (dark green). Specificity for the proximal domain is regulated by dFezf (not shown). An additional lamina neuron subclass (blue-green) terminates in a distal domain of the outer medulla. (**c**) (left panel) L3 growth cones undergo a stereotyped structural rearrangement that segregates them into the developing target layer. Another lamina neuron (dark green) forms an additional arborization in the distal outer medulla. These events contribute to the emergence of discrete layers. (middle and right panels) DFezf activates *Netrin* expression in L3 neurons, and Netrin (purple) is secreted from L3 growth cones (green) providing an M3-specific cue for R8 photoreceptor growth cones (red). The sequential targeting of L3 and R8 growth cones contributes to M3 development. (**d**) Within the target layer, L3 axons (green) may distinguish between appropriate (dark orange) and inappropriate (light orange) synaptic targets through specific cell recognition molecules such as Dpr and Dip proteins

The studies described above imply a dynamic mode of layer assembly in the medulla, wherein layers form from broad regions in a stepwise manner during development through a precise sequence of interactions between specific cell types (see Fig. 6). Cellular processes within the nascent outer medulla, inner medulla and serpentine layer express repulsive and adhesive cell surface molecules in a complementary manner. These molecules regulate targeting to the outer and inner medulla, potentially in conjunction with the timing of medulla innervation (Fig. 6a). Within the early outer medulla, axons initially target in an overlapping manner establishing broad domains (Fig. 6b). Within these domains, specific layers develop through a process of addition and subtraction, as neurites undergo local rearrangements, form additional arborizations, and become refined to achieve their mature morphologies (Fig. 6c). Interactions between both synaptic and non-synaptic partners contribute to layer refinement, and transcriptional mechanisms (e.g. dFezf) are dedicated to coordinating the layer innervation of different neuron types. Finally, within layers, the complementary expression of cell adhesion molecules belonging to specific gene families in appropriate synaptic partners may regulate synaptic specificity (Fig. 6d). Dpr and DIP Ig proteins are particularly intriguing candidates due to their binding specificities and matching patterns of expression in pre- and postsynaptic neurons.

Research in other systems has suggested that neurites innervate specific layers or positions through a “layer code”, defined by molecular gradients or homophilic cell adhesion molecules. This implies that layers in these systems are pre-patterned prior to neurite innervation, and serve as a template for circuit formation. For example, in the *Drosophila* embryonic ventral nerve cord different types of sensory axons terminate and branch at characteristic positions within each abdominal hemisegment amidst a dense assortment of cellular processes. Zlatic and colleagues argued that, in this context, neurite positioning is pre-defined by molecular gradients that act globally on incoming processes to instruct neurite targeting [77, 78]. Similarly, in the zebrafish optic tectum it has been proposed that gradients of repulsive and adhesive molecules position retinal ganglion cell axons and tectal dendrites within specific laminae [79, 80]. Interestingly, in both systems glia are thought to be the cellular source of molecular gradients. Within the inner plexiform layer (IPL) of the chick retina wherein different bipolar, amacrine and ganglion cells confine their neurites to specific sublaminae and form connections, Yamagata and colleagues discovered that homophilic proteins of the Ig superfamily are expressed in non-overlapping populations of cells and within specific sublaminae [81–83]. Loss and gain of function experiments revealed that these molecules are necessary and sufficient for laminar specificity. Based on this, it was proposed that synaptic partners are matched to target sublaminae by a code of homophilic Ig protein expression. However, how Ig proteins are arranged in a sublaminae-specific manner within the early IPL is unclear. In these models, different neurons innervate the same position or layer through a common mechanism, either by commonly expressing the same levels or types of guidance receptors, or by expressing the same homophilic Ig domain-containing cell adhesion molecules. In the latter example, homophilic interactions between synaptic partners could regulate laminar specificity and synaptic specificity.

By contrast, in the medulla layers are not pre-established, and different neurons innervate the same layers through different molecular mechanisms. For instance, while L5 neurons depend on CadN for innervating the M2 layer, CadN is dispensable in L2 neurons for layer specificity [36, 62]. In addition, R8 neurons depend on Netrin to innervate the M3 layer, but L3 layer specificity is independent of Netrin function (M.Y.P. unpublished), as is the dendritic targeting of Tm9 neurons (M.Y.P. unpublished), which are postsynaptic to both L3 [57, 58, 84] and R8 [84] axons within the M3 layer. While the mechanisms that underlie layer specificity in these neurons are yet to be fully characterized, these findings argue against the existence of a “layer code” in the medulla, in which each cell type innervating the same layer uses a common mechanism to do so. Some neurons in the medulla may utilize layer-specific cues to innervate layers (e.g. R8 targeting depends on M3-specific Netrin localization), particularly at late stages of development when layers are refined. However, these cues are likely to function in a temporal and cell type-specific manner.

In the absence of a “layer” code, how do neurons in the medulla know which layers to innervate? Based on our finding that dFezf orchestrates M3 assembly by regulating the stepwise targeting of L3 and R8 axons, we propose that part of the answer lies in the use of transcriptional modules to coordinate the layer innervation of specific cell types. In the lamina each lamina neuron subtype (L1-L5) uniquely expresses a specific transcription factor [68] (L3 neurons selectively express dFezf), and lamina neuron arborizations within developing medulla layers are well positioned to release cues (like Netrin) that recruit other cell types. Thus, similar to dFezf in L3 neurons, transcription factors specific to other lamina neurons may function to cell-intrinsically direct targeting to a developing layer, and cell extrinsically recruit particular neuron types.

### Conserved mechanisms for building synaptic layers?

The medulla is analogous to the vertebrate IPL in structure and function [85]. While discrete sublaminae in the chick IPL may be established through a code of homophilic Ig proteins, research in the mouse IPL suggests an alternative mechanism that is reminiscent of layer development in the medulla. The IPL is organized into OFF and ON regions based on the physiological and targeting properties of bipolar cells, which are analogous to lamina neurons. Bipolar cells that are activated by light decrements stratify in distal sublaminae (OFF), and bipolar cells that are activated by light increments innervate proximal sublaminae (ON). Matsuoka and colleagues found that, in the mouse IPL, PlexinA4 (PlexA4) is selectively expressed in ON sublaminae, while its ligand Semphorin6A (Sema6A) is concentrated in OFF sublaminae [86]. Disruption of Sema6A/PlexA4 signaling caused amacrine cells that normally innervate OFF sublaminae to inappropriately innervate ON sublaminae. This is reminiscent of how disruption of Sema-1a/PlexA signaling in the medulla causes lamina neurons that normally innervate the outer medulla to inappropriately target to the inner medulla [62]. As PlexA4 and Sema6A are expressed in a complementary pattern within the early IPL, it was proposed that PlexA4/Sema6A signaling regulates the initial targeting of processes to broad domains. Thus, similar to medulla layers, sublaminae within the mouse IPL may form dynamically from broad regions during development.

Interestingly, Fezf1 and 2 have been shown to be expressed in a subset of OFF bipolar cells in the mouse retina [87]. As L3 neurons, which express dFezf, are necessary for OFF-edge motion detection [88], this indicates that Fezf transcription factors are expressed in similar cell types that innervate analogous structures in the mouse retina and fly visual system. Given that dFezf plays a central role in regulating layer assembly in the medulla, Fezf1 and 2 may similarly orchestrate laminar-specific connectivity in the IPL.

Fezf2 has been shown to play a key role in layer assembly within the mouse cerebral cortex. Within this brain region, specific types of pyramidal neurons and inhibitory neurons become integrated into layer-specific circuits. Fezf2 is selectively expressed in subcortically projecting pyramidal neurons (subcerebral projection neurons) that are predominantly localized to layer V, and is cell autonomously required for the specification of these neurons [89–91]. In the absence of Fezf2 function, these neurons are absent from the cortex, and when mis-expressed Fezf2 has the intrinsic ability to impart a subcerebral projection neuron identity to cells that would otherwise differentiate into alternative neuron types [92–94]. Thus Fezf2 cell-intrinsically instructs subcerebral projection neuron identity. Lodato and colleagues found that the identity of pyramidal neurons plays an instructive role in the laminar positioning of inhibitory neurons [95]. For example, the generation of subcerebral projection neurons at abnormal locations within the cortex via ectopic expression of Fezf2, was sufficient to recruit the appropriate types of inhibitory neurons. It remains unclear if Fezf2 regulates the expression of factors (e.g. secreted molecules) that non-autonomously control the layer positions of specific inhibitory neurons. However, one interpretation of these findings is that Fezf2 in the cortex coordinates the assembly of layer-specific circuits through cell-intrinsic and cell-extrinsic mechanisms, analogous to dFezf in the medulla.

Collectively, these findings appear to hint at evolutionarily shared mechanisms for building layered networks of neural connections.

### Are common strategies used to organize circuits in layered and non-layered regions?

Comparison of the mechanisms giving rise to layers in the medulla and glomeruli within the *Drosophila* antennal lobe, suggest that both common and distinct strategies underlie circuit formation in these regions.

Within the antennal lobe, connections between olfactory sensory neurons (OSNs) and second order projection neurons (PNs) are concentrated in structurally discrete glomeruli. Within each glomerulus a single class of OSNs expressing the same olfactory receptor synapses onto a single type of PN [96–102]. Glomeruli within the antennal lobe arise in a stepwise manner during metamorphosis (reviewed in [103]). Early in pupal development PN dendrites innervate the developing antennal lobe and segregate into course domains [104] through a combination of repulsive and adhesive interactions. Interactions between the Sema-1a receptor, expressed by PNs, and the Sema-2a/2b ligands, which are secreted by larval olfactory sensory neurons, induces repulsion [105, 106]. It was suggested that PNs express different levels of Sema-1a and this causes different PNs to experience different levels of repulsion, causing their dendrites to become differentially distributed within the antennal lobe. In addition, CadN-mediated adhesion, potentially between PNs of the same class, also restricts dendrite branching to particular domains [107]. Within course domains, cell surface molecules expressed in a class-specific manner (e.g. the leucine rich repeat protein capricious [108]) instruct the segregation of neighboring PNs into class-specific glomeruli. Subsequently, OSN axons project into the antennal lobe and target to course positions based on axon-axon interactions [109] (Semaphorin proteins), by responding to secreted target-derived cues (e.g. hedgehog [110]), and through additional mechanisms [111–113]. Within these course regions, selective cell-cell interactions with PNs (in part mediated by homophilic Teneurin molecules [114]) control innervation of specific glomeruli [115, 116].

Analogous to how layers develop in the medulla, glomeruli emerge progressively from initially broad regions through a precise order of cell-cell interactions. Strikingly, in both the early medulla and early antennal lobe, adhesive and repulsive interactions mediated by CadN and Semaphorin proteins act in combination to restrict innervation to course regions. In addition, within course regions discrete layers or glomeruli are refined through local interactions between specific cell types. Interestingly, a key step in the development of discrete glomeruli is the pre-positioning of PN dendrites within the antennal lobe. This defines glomerular position and provides precise targeting instructions for OSN axons, which innervate the antennal lobe later in development. Thus, the formation of discrete glomeruli appears to be controlled through a combination of stepwise refinement and template-based mechanisms. Within the medulla, it is unlikely that a general pre-patterning mechanism is utilized following the establishment of broad domains to provide precise targeting coordinates for incoming processes. Particular neurons depend on other neurons for layer-specific cues, however these mechanisms appear to be cell type-specific rather than layer-specific. For example, L3-derived Netrin is necessary for the M3-specific innervation of R8, but Tm9 neurons innervate M3 independent of L3 [64]. This could reflect the fact that many more neuron types form connections within medulla layers than within glomeruli in the antennal lobe.

### Open questions

Despite the recent advances in understanding how medulla layers are established many open questions remain. For example, the organization of neural processes into broad domains within the early medulla is crucial for the proper development of specific layers, yet molecular and cellular mechanisms that regulate broad domain specificity remain poorly understood. In addition, while layer specificity does not appear to be determined by a “layer code”, how the precision of layer innervation is regulated in specific cell types has not been addressed. Another limitation to our knowledge of layer assembly in the medulla is that, up to this point, layer specificity has been predominantly studied from the point of view of photoreceptors and lamina neurons, and how their synaptic targets innervate specific layers is largely unknown. Moreover, the extent to which neural activity and genetic mechanisms interact to regulate layer-specific connectivity, and how synaptic specificity within layers is achieved are unknown.

Given that layers in the medulla are established via a process of self-assembly, involving a choreographed sequence of interactions during development, the initial cellular interactions that give rise to the nascent medulla provide the foundation on which discrete layers are built. Thus, to elucidate the molecular and cellular logic underlying assembly of the medulla network it is crucial to (1) identify the cellular and molecular underpinnings of early medulla organization, (2) address the mechanisms governing the series of interactions giving rise to specific layers, and (3) identify commonalities or connections between the formation of different layers. While this is a monumental task that would not be achievable in most complex systems, the stereotyped architecture of the medulla and the ever-increasing number of tools for genetically manipulating specific cell types in this system provide a unique opportunity to address this.

### Concluding remarks

To assemble into regularly spaced columnar and layered networks neurons must identify correct synaptic targets amidst numerous alternatives. Research in the *Drosophila* visual system has illuminated developmental, molecular and cellular strategies that underlie how neurons accomplish this and integrate into the appropriate circuits with high fidelity and precision.

Significant overlap exists between the strategies and molecules used to construct columns and layers in flies, and similar strategies are employed to build neural circuits in mammals. In addition, there are striking similarities between how columns and layers and non-columnar/layered circuits are assembled, suggesting common rules govern the formation of neural circuits regardless of their structure.

## Abbreviations

babo: Baboon
CadN: N-Cadherin
Dip: dpr interacting protein
Dpr: Defective proboscis response
EM: electron microscopy
Fmi: Flamingo
h APF: hours after puparium formation
Imp-α3: Importin-α3
IPL: inner plexiform layer
MeT: Medulla tangential cell
Mi: medulla intrinsic neuron
Pcdh: Protocadherin
PlexA: PlexinA
PlexA4: PlexinA4
Sema-1a: Semaphorin-1a
Sema-6A: Semaphorin-6A
Tm: transmedullary neuron
TmY: transmedullary Y neuron
Tutl: Turtle
